# Simple Novel Screening Tool for Obstructive Sleep Apnea in Inflammatory Bowel Disease

**DOI:** 10.1093/crocol/otad016

**Published:** 2023-03-17

**Authors:** Alex Barnes, Jane M Andrews, Sutapa Mukherjee, Robert V Bryant, Peter Bampton, Paul Spizzo, Robert J Fraser, Réme Mountifield

**Affiliations:** Department of Gastroenterology, Southern Adelaide Local Health Network (SALHN) Flinders Medical Centre, Bedford Park, South Australia, Australia; Adelaide Institute for Sleep Health, Flinders Health and Medical Research Institute, College of Medicine and Public Health, Flinders University, Bedford Park, South Australia, Australia; Inflammatory Bowel Disease Service, Department of Gastroenterology and Hepatology, (CAHLN) Royal Adelaide Hospital, Adelaide, South Australia, Australia; Faculty of Health & Medical Sciences, School of Medicine, University of Adelaide, Adelaide, South Australia, Australia; Adelaide Institute for Sleep Health, Flinders Health and Medical Research Institute, College of Medicine and Public Health, Flinders University, Bedford Park, South Australia, Australia; Department of Respiratory and Sleep Medicine, Southern Adelaide Local Health Network (SALHN) Flinders Medical Centre, Bedford Park, South Australia, Australia; Faculty of Health & Medical Sciences, School of Medicine, University of Adelaide, Adelaide, South Australia, Australia; Department of Gastroenterology, Queen Elizabeth Hospital, Woodville, South Australia, Australia; Adelaide Institute for Sleep Health, Flinders Health and Medical Research Institute, College of Medicine and Public Health, Flinders University, Bedford Park, South Australia, Australia; Department of Gastroenterology, Southern Adelaide Local Health Network (SALHN) Flinders Medical Centre, Bedford Park, South Australia, Australia; Department of Gastroenterology, Southern Adelaide Local Health Network (SALHN) Flinders Medical Centre, Bedford Park, South Australia, Australia; Adelaide Institute for Sleep Health, Flinders Health and Medical Research Institute, College of Medicine and Public Health, Flinders University, Bedford Park, South Australia, Australia; Department of Gastroenterology, Southern Adelaide Local Health Network (SALHN) Flinders Medical Centre, Bedford Park, South Australia, Australia; Adelaide Institute for Sleep Health, Flinders Health and Medical Research Institute, College of Medicine and Public Health, Flinders University, Bedford Park, South Australia, Australia

**Keywords:** inflammatory bowel disease, obstructive sleep apnea, disability, psychology

## Abstract

**Background:**

Inflammatory bowel disease (IBD) has been associated with an increased risk of obstructive sleep apnea (OSA). We aimed to examine the associations of obstructive sleep apnea, sleepiness, and IBD-related data and comorbidities, with the aim of developing a screening tool for sleep apnea in this population.

**Methods:**

An online survey of adults with IBD was administered which included measures of assessment of the risk of OSA, and measures of IBD activity, IBD-related disability, anxiety, and depression. Logistic regression was performed to investigate the associations between the risk of OSA and IBD data, medications, demographics, and mental health conditions. Further models were built for an outcome of severe daytime sleepiness and a combined outcome of risk of OSA and at least mild daytime sleepiness. A simple score was constructed for the purpose of screening for OSA.

**Results:**

There were 670 responses to the online questionnaire. The median age was 41 years, the majority had Crohn’s disease (57%), the median disease duration was 11.9 years, and approximately half were on biologics (50.5%). Moderate–high risk of OSA was demonstrated in 22.6% of the cohort. A multivariate regression model for moderate–high risk of OSA included increasing age, obesity, smoking, and abdominal pain subscore. For a combined outcome of moderate–high risk of OSA and at least mild daytime sleepiness, a multivariate model included abdominal pain, age, smoking, obesity, and clinically significant depression. A simple score was constructed for screening for OSA utilizing age, obesity, IBD activity, and smoking status with an area under the receiver-operating curve of 0.77. A score >2 had a sensitivity of 89% and a specificity of 56% for moderate–high risk of OSA and could be utilized for screening for OSA in the IBD clinic.

**Conclusions:**

Over one-fifth of an IBD cohort met significantly high-risk criteria for OSA to warrant referral for a diagnostic sleep study. The risk of OSA was associated with abdominal pain, along with more traditional risk factors such as smoking, increasing age, and obesity. Consideration should be given for screening for OSA in IBD patients utilizing a novel screening tool that utilizes parameters typically available in IBD clinic.

## Introduction

Inflammatory bowel disease (IBD) is a chronic relapsing–remitting inflammatory condition that is increasing in frequency worldwide.^1^ The associated gastrointestinal symptoms may disrupt sleep leading to poor sleep quality. The prevalence of poor sleep in this population has been reported in a recent meta-analysis to be 56%.^2^ Poor sleep is more common in those with IBD than controls,^3^ more common in those with active IBD than inactive IBD,^3,4^ and more common in those with inactive IBD than controls.^5^ Mental health conditions such as anxiety and depression have been associated with poor sleep in an IBD population.^6–13^

Although IBD-associated upper airway obstruction is rare,^14,15^ obstructive sleep apnea (OSA) was shown to be more common in people with IBD in a study utilizing US-wide diagnostic coding data.^16^ This finding was supported by a previous study that utilized an online screening questionnaire in a UK population.^17^

OSA is associated with a variety of medical conditions including obesity, cardiovascular disease,^18,19^ Parkinson’s disease,^20^ and gastro-esophageal reflux disease.^21,22^ Relevant to IBD, OSA has been associated with an increase in circulating tumor necrosis factor-alpha (TNF-a) levels, with higher levels associated with more severe obstruction and hypoxia.^23^ Anti-TNF-a therapy has been associated with improved sleepiness in obese patients with OSA^24^ and a lower frequency of OSA spondyloarthropathy population.^25^ Active IBD is associated with elevated TNF-a levels and consequently may influence the course or development of OSA. Furthermore, obesity is prevalent in people with IBD^26–28^ and may somewhat explain the observed increased rates of sleep apnea. However, in a nationwide study in a US population, IBD remained associated with OSA after controlling for known risk factors such as obesity.^16^ This suggests that risk factors for OSA in an IBD population may be different from known traditional risk factors.

Our study aimed to examine the rates of OSA and sleepiness in an IBD population as well as examine factors associated with OSA and sleepiness. The study also aimed to construct a simple score for screening for OSA using typical IBD clinic parameters.

## Methods

Ethics approval for this study was obtained from the Southern Adelaide Human Research Ethics Committee (203.20). An online questionnaire was distributed to individuals with IBD through tertiary hospital IBD clinic patient email lists, a private gastroenterology practice patient email list, and through Crohn’s Colitis Australia, a charity organization, via email advertising and social media. Tertiary hospital IBD units and private gastroenterology groups routinely collect patient email addresses to allow communication between the IBD unit and the IBD cohort under care. Individuals with a self-reported diagnosis of IBD over 18 years of age were asked to participate. Demographic data such as age and sex were recorded, along with data on IBD which included disease duration, previous surgery, and current medications.

The OSA-50 is a validated screening tool for OSA in primary care.^29,30^ It contains 4 components with scores for waist circumference, age, snoring, and observed cessation of breathing during sleep. An OSA-50 score over 5 has been associated with moderate–severe OSA and is sufficient to justify direct referral for a sleep study in Australia (sensitivity 94%).^29^ The Epworth Sleepiness Scale (ESS) is a validated measure of daytime sleepiness.^31^ A score below 6 is considered below normal sleepiness, 6–10 is normal, 11–12 is mild sleepiness, 13–15 is moderate sleepiness, and a score over 15 is considered to represent severe daytime sleepiness. The ESS in combination with the OSA-50 has been shown to provide high specificity for OSA (94%), however, with reduced sensitivity (51%).^32^

IBD disease activity was assessed using the modified Harvey Bradshaw Index (HBI) in the case of Crohn’s disease with HBI > 5 considered an active disease,^33^ excluding the physical exam question. The Simple Clinical Colitis Activity Index (SCCAI) was utilized to assess disease activity in those with ulcerative colitis, with an SCCAI > 2 considered active disease.^34^ The abdominal pain subscore from HBI was utilized to form an abdominal pain dichotomous variable with an abdominal pain subscore of mild used as the cutoff value, with values above mild encoded as 1, and values mild or below encoded as a 0. The nocturnal diarrhea subscore from SCCAI was utilized to form a nocturnal diarrhea dichotomous variable with a nocturnal diarrhea subscore of >1 encoded as a 1, and scores 1 or less encoded as a 0.

Anxiety was assessed using the Generalized Anxiety Disorder 7-Item Scale (GAD-7)^35^ with a score over 10 used to indicate likely clinically significant anxiety. The Patient Health Questionnaire-9 (PHQ-9) was used to assess depression with a score of >15 used to indicate likely clinically significant depression.^36^

Disability is defined as any inability to perform an activity considered normal for a human.^37^ Disability was assessed using the IBD disability index self-report (IBD-DI-SR).^38^ The IBD-DI-SR is a validated self-reported measure of disability in an IBD population. It was developed as a self-report form and a short form of the IBD disability index.^39^

Statistical analysis was performed using Stata SE 16 (StataCorp). For normally distributed variables, mean and standard deviation were reported with comparisons made using the student *t-*test. For non-normally distributed variables, median and interquartile range (IQR) were reported, with comparisons made using the Mann–Whitney *U-*test. For categorical data, Pearson’s *χ*^2^ test was used, or Fisher’s exact test when appropriate. Univariate logistic regression was performed, and a multivariate logistic regression model was built for moderate–high risk of OSA (OSA-50 > 5) incorporating all variables from univariate analysis with *P* < .10. The multivariate model was optimized by sequentially adding and removing variables to maximize the likelihood function. Separate models were constructed for an outcome of severe daytime sleepiness (ESS > 15) and for a combined outcome of moderate–high risk of OSA (OSA-50 > 5) and at least mild daytime sleepiness (ESS > 10). Variables common to the multivariate models and considered to be typically available in IBD clinic were then further analyzed to create a score for screening for OSA.

## Results

There were 670 responses to the online questionnaire. The median age was 41 years (32–70), with most being female (78%). The majority had Crohn’s disease (57%). The mean disease duration was 11.9 years (10.4), 30% had undergone surgery for IBD, and around half were on biologics (50.5%; see Table 1). Clinically significant depression (PHQ-9 > 15) was seen in 18%, and clinically significant anxiety (GAD-7 > 10) was seen in 29%. The mean IBD-DI-SR was −2.78 (6.01).

**Table 1.** Demographic, IBD-related data, medications, clinical IBD activity, mental health conditions and disability scores of the cohort.

	Cohort	OSA-50 > 5	OSA-50 ≤ 5	*P* value
Age, median IQR	41 (32–70)	49.9 (47.8–52.1)	40.3 (39.1–41.5)	<.001
Female gender, *n* (%)	525 (78)	73%	78%	.266
Crohn’s disease, *n* (%)	384 (57)	59%	61%	.59
Disease duration years, mean (SD)	11.9 (10.4)	13.8 (11.9–15.7)	12.2 (11.2–13.1)	.095
Previous surgery for IBD, *n* (%)	201 (30.0)	72%	67%	.24
Current steroid use, *n* (%)	58 (8.6)	11%	9%	.30
Current biologic use, *n* (%)	339 (50.5)	56%	52%	.41
Current immunomodulator use, *n* (%)	228 (34.0)	39%	36%	.41
Obesity, *n* (%)	247 (36.9)	66%	26%	<.001
Smoking, *n* (%)	44 (6.6)	12%	5%	.004
Alcohol usage, *n* (%)	213 (31.8)	36%	33%	.13
Opioid usage, *n* (%)	93 (13.9)	20%	14%	.081
Medications for sleep, *n* (%)	85 (12.7)	14%	14%	.95
Clinically significant depression, *n* (%)	120 (17.9)	31%	23%	0.069
Clinically significant anxiety, *n* (%)	193 (28.8)	32%	36%	.37
Clinically active IBD SCCAI, mean (SD) HBI, mean (SD)	7.18 (2.86) 7.08 (3.29)	7.90 (7.42–8.39) 7.70 (7.13–8.28)	6.95 (6.68–7.23) 6.89 (6.58–7.20)	.0010 .013
IBD-DI-SR, mean (SD)	−2.78 (6.01)	−4.91 (−5.56 to −3.94)	−3.04 (−3.65 to −2.51)	.0008

Results reported for OSA-50 score over 5, with *P* value using Student’s *t*-test or Pearson’s *χ*^2^ as appropriate. OSA-50 is a screening test for obstructive sleep apnea. HBI—Harvey Bradshaw Index measures clinical activity of Crohn’s disease. SCCAI—simple clinical colitis activity index measures clinical activity of ulcerative colitis. IBD-DI-SR is a self-reported questionnaire that measures disability in an IBD population. Clinically significant anxiety based on a Generalized Anxiety Scale-7 score greater than 10. Clinically significant depression based on a Patient Health Questionnaire-9 score over 15.

Abbreviations: IBD, inflammatory bowel disease; IQR, interquartile range; OSA, obstructive sleep apnea; SD, standard deviation.

The median OSA-50 score was 3, with 22.6% having an OSA-50 score over 5 (see Table 1). Those who had an OSA-50 score over 5 were older (*P* < .001), smokers (*P* = .004), obese (*P* < .001), and had higher IBD activity scores (*P* = .001, and *P* = .013). Worse disability scores were seen in those with an OSA-50 score over 5 (*P* = .0008).

Univariate and multivariate logistic regressions for an outcome of moderate–high risk of OSA were performed including demographics, IBD data, IBD medications, and IBD clinical activity (see Table 2). Univariate regression was significant for increasing age, obesity, clinically significant depression, abdominal pain subscore, nocturnal diarrhea subscore, and clinically active IBD. A multivariate model was optimized with the final model including increasing age, obesity, smoking, and abdominal pain subscore (see Table 2). Male gender was not significant. Male gender was associated with lower rates of obesity (*P* = .002), lower rates of clinically significant depression (*P* = .004), and lower rates of clinically active IBD (*P* < .001) but was also associated with higher rates of smoking (*P* = .049), and males were on average older (45.8 years [43.3–48.4] vs 41.8 [40.7–42.9], *P* = .0013).

**Table 2.** Univariate and multivariate regressions for moderate–high risk of sleep apnea (OSA-50 > 5) considering demographic data, IBD medications, depression, anxiety, and IBD data.

	Univariate regression (odds ratio, confidence interval, *P* value)	Multivariate regression (odds ratio, confidence interval, *P* value)
Age	1.05 (1.04–1.07), *P* < .001	1.06 (1.04–1.08), *P* < .001
Male gender	1.28 (0.83–1.99), *P* = .27	
Crohn’s disease	0.90 (0.61–-1.33), *P* = .60	
Disease duration	1.01 (0.99–1.03), *P* = .096	
Previous surgery for IBD	1.28 (0.84–1.96), *P* = .24	
Current steroid use	1.38 (0.75–2.54), *P* = .302	
Current biologic use	1.17 (0.80–1.72), *P* = .41	
Current immunomodulator use	1.18 (0.79–1.74), *P* = .42	
Obesity	5.39 (3.58–8.10), *P* < .001	6.42 (4.08–10.10), *P* < .001
Smoking	2.52 (1.31–4.81), *P* = .005	2.60 (1.26–5.39), *P* = .010
Alcohol usage	1.16 (0.78–1.73), *P* = .45	
Opioid usage	1.55 (0.94–2.55), *P* = .082	
Medications for sleep	0.98 (0.57–1.70), *P* = .95	
Clinically significant depression	1.76 (1.13–2.75), *P* = .013	
Clinically significant anxiety	0.92 (0.61–0.139), *P* = .69	
Abdominal pain subscore	1.33 (1.09–1.62), *P* = .004	1.32 (1.05–1.65), *P* = .017
Nocturnal diarrhea subscore	2.11 (1.11–3.99), *P* = .022	
Clinically active IBD	1.73 (1.01–2.93), *P* = .044	

Abbreviations: IBD, inflammatory bowel disease; OSA, obstructive sleep apnea.

The mean ESS score was 7.9 (4.75), with 13.4% describing severe daytime sleepiness. Severe daytime sleepiness was associated with worse disability scores (*P* = .0001). Univariate and multivariate logistic regressions were performed including demographics, IBD data, IBD medications, and IBD clinical activity (see Table 3). Univariate regression was significant for smoking, clinically significant depression, clinically significant anxiety, and abdominal pain subscore. Multivariate regression was significant for clinically significant depression, clinically significant anxiety, and abdominal pain subscore (see Table 3).

**Table 3.** Univariate and multivariate regressions for significant sleepiness (Epworth Sleepiness Score >14) considering demographic data, IBD medications, depression, anxiety, and IBD data.

	Univariate regression (odds ratio, confidence interval, *P* value)	Multivariate regression (odds ratio, confidence interval, *P* value)
Age	0.99 (0.97–1.01), *P* = .23	
Male gender	0.87 (0.49–1.54), *P* = .64	
Crohn’s disease	1.03 (0.64–1.67), *P* = .88	
Disease duration	0.99 (0.97–1.01), *P* = .46	
Previous surgery for IBD	0.95 (0.58–1.56,) *P* = .84	
Current steroid use	1.66 (0.82–3.36), *P* = .16	
Current biologic use	0.92 (0.58–1.47), *P* = .75	
Current immunomodulator use	0.96 (0.59–1.56), *P* = .87	
Obesity	1.41 (0.88–2.26), *P* = .15	
Smoking	2.48 (1.20–5.16,) *P* = .015	
Alcohol usage	0.88 (0.54–1.45), *P* = .63	
Opioid usage	1.17 (0.63–2.18), *P* = .62	
Medications for sleep	0.84 (0.41–1.70), *P* = .63	
Clinically significant depression	3.94 (2.38–6.53), *P* < .001	2.65 (1.46–4.80), *P* = .001
Clinically significant anxiety	2.99 (1.86–4,84), *P* < .001	1.66 (0.94–2.96), *P* = .082
Abdominal pain subscore	1.42 (1.13–1.78), *P* = .002	1.33 (1.04–1.69), *P* = .021
Nocturnal diarrhea subscore	1.66 (0.82–3.36), *P* = .16	
Clinically active IBD	1.69 (0.87–3.30), *P* = .12	

Abbreviations: IBD, inflammatory bowel disease; OSA, obstructive sleep apnea.

A combined outcome of moderate–high risk of OSA (OSA > 5) and at least mild daytime sleepiness (ESS > 11) was considered. Univariate regression was significant for age, current corticosteroid use, obesity, current smoking, clinically significant depression, abdominal pain, and clinically active IBD (see Table 4). Multivariate regression was significant for age, obesity, smoking, clinically significant depression, and abdominal pain (see Table 4).

**Table 4.** Univariate and multivariate regression for combined outcome of moderate–high risk of sleep apnea (OSA-50 > 5) and at least mild sleepiness (ESS > 11) considering demographic data, IBD medications, depression, anxiety, and IBD data.

	Univariate regression (odds ratio, confidence interval, *P* value)	Multivariate regression (odds ratio, confidence interval, *P* value)
Age	1.03 (1.01–1.05), *P* = .001	1.03(1.01–1.06), *P* = .002
Male gender	1.25 (0.68–2.29), *P* = .47	
Crohn’s disease	0.86 (0.50–1.47), *P* = .59	
Disease duration	0.99 (0.97–1.02), *P* = .60	
Previous surgery for IBD	1.44 (0.78–2.64), *P* = .24	
Current steroid use	2.12 (1.01–4.45), *P* = .046	
Current biologic use	1.12 (0.70–2.04), *P* = .50	
Current immunomodulator use	1.47 (0.86–2.51), *P* = .16	
Obesity	5.29 (2.96–9.43), *P* < .001	5.25 (2.86-9.64), *P* < .001
Smoking	3.21 (1.49–6.91), *P* = .003	3.33 (1.44–7.67), *P* = .005
Alcohol usage	0.82 (0.46–1.47), *P* = .52	
Opioid usage	1.46 (0.74–2.86), *P* = .27	
Medications for sleep	1.26 (0.61–2.58), *P* = .53	
Clinically significant depression	2.14 (1.24–3.70), *P* = .006	1.91 (1.04–3.48), *P* = .036
Clinically significant anxiety	1.55 (0.90–2.63), *P* = .11	
Abdominal pain subscore	1.44 (1.12–1.84), *P* = .004	1.37 (1.07–1.76), *P* = .011
Nocturnal diarrhea subscore	2.26 (0.96–5.33), *P* = .061	
Clinically active IBD	3.89 (1.38–10.96), *P* = .010	

Abbreviations: IBD, inflammatory bowel disease; ESS, Epworth Sleepiness Score; OSA, obstructive sleep apnea.

A simple score was constructed (see Table 5) for the risk of OSA utilizing variables typically available in IBD clinic. The area under the receiver-operating curve for moderate–high risk of OSA (OSA-50 > 5) was 0.77 (0.73–0.81), with Youden’s index of 1.46. A score of >2 had a sensitivity of 89% and a specificity of 56%. The area under the receiver-operating curve for moderate–high risk of OSA (OSA-50 > 5) and at mild daytime sleepiness (ESS > 10) was 0.77 (0.71–0.82), with Youden’s index of 1.41. A score of >2 had a sensitivity of 89% and a specificity of 56% for moderate–high risk of OSA.

**Table 5.** Simple score for screening for obstructive sleep apnea utilizing variables commonly available in IBD clinic—obesity (2 points), current smoking (1 point), age over 45 (1 point), and clinically active IBD (1 point).

Variables	Score, if present
Obesity	2
Currently smoking	1
Age over 45	1
Clinically active IBD	1

The area under the receiver-operating curve for moderate–high risk of obstructive sleep apnea (OSA-50 > 5) was 0.77 (0.73–0.81), with Youden’s index of 1.46. A score of >2 had a sensitivity of 89% and a specificity of 56%. Obesity is defined as body mass index greater than 30, clinically active IBD refers to a Harvey–Bradshaw Index of >5 or a Simple Clinical Colitis Activity Index of >2.

Abbreviations: IBD, inflammatory bowel disease; OSA, obstructive sleep apnea

## Discussion

In a large IBD cohort, the rate of and novel associations with OSA have been described. Over one-fifth of the cohort met the criteria for high risk of OSA and could on this basis be referred for diagnostic polysomnography. The risk of OSA was associated with increased age, obesity, smoking, and abdominal pain in a cohort of people with IBD. We also explored sleepiness, a common symptom of sleep apnea, with this associated in our cohort with mental health conditions and abdominal pain. We also proposed a novel simple score incorporating parameters typically used in IBD clinic to identify those at risk of OSA. The score incorporated parameters typically available in IBD clinic and could consequently be readily applied.

Well-established risk factors for OSA, including older age,^40–42^ smoking,^43^ and obesity^44^, were apparent in our data; however, no association with male gender,^40,45,46^ as previously well described with OSA, was seen. This may be a result of the female predominance of the cohort (78% female), noting that a previous study did note an association between male gender and OSA in an IBD cohort.^16^ We also note the perhaps confounding relationship between gender and other risk factors for OSA in our data set such as IBD activity and depression.

Abdominal pain was associated with OSA and sleepiness. Poor sleep has been associated with an increased perception of pain which may partly explain these results.^47,48^ Furthermore, OSA has been associated with irritable bowel syndrome^49^—known to be common in those with IBD.^50–52^ In addition, OSA-related nocturnal hypoxia may contribute to localized intestinal ischemia that has been previously postulated to play a role in the pathogenesis of IBD.^53^

OSA has been associated with cardiovascular disease^54^ and, in particular, stroke,^55–57^ which has been attributed to systemic inflammation and as well apnea-induced nocturnal ischemia.^58–60^ Identification of those with OSA will allow screening for associated cardiovascular complications and commencement of treatment such as continuous positive airway pressure (CPAP).^61^ Treatment of those with OSA is associated with improved daytime sleepiness, along with improved quality of life.^62^ Treatment of OSA has been shown to reduce blood pressure in those with hypertension^63^ and additionally, long-term observational data also suggest a reduction in ischemic heart disease and fatal cardiac events with the usage of CPAP,^64^ although no overall mortality benefit has been demonstrated and randomized controlled trial results have been mixed.^65^ IBD has been associated with increased risk of cardiovascular disease^66,67^ and consequently, consideration should be given to identifying and treating those with OSA to reduce cardiovascular risk. The OSA-50 is commonly used to screen for OSA in Australia; however, this incorporates parameters typically not available in IBD clinic such as apneic events, waist circumference, and snoring. We proposed a simple score incorporating parameters available in IBD clinic which could be used to screen for OSA and is consequently perhaps much more attractive to gastroenterologists. Further validation of this score in other IBD cohorts is required.

Limitations of this study include selection bias due to the use of an online questionnaire and the predominantly female cohort. The rates of anxiety and depression seen in our cohort were similar to that described elsewhere.^68^ Our cohort likely represents a moderate–severe IBD cohort with biologic usage in around half and previous surgery in almost a third. Given that OSA is more common in males, the prevalence of moderate–high risk of OSA seen here is likely lower than in the broader IBD population.^69^ Reporting bias may also be significant, noting a study of people with Crohn’s disease reported worse sleep quality than that observed by objective measures.^70^ The absence of objective measures of sleep quality and objective IBD activity is also considered a limitation. Further studies should consider objective measures of IBD activity and sleep quality.

## Conclusions

Over one-fifth of an IBD cohort met high-risk criteria for OSA to warrant referral for a diagnostic sleep study. Risk of OSA was associated with abdominal pain, along with more traditional risk factors such as smoking, increasing age, and obesity. Those at risk of OSA have worse disability scores. A simple score using typical IBD clinic data could be used to screen for OSA.

## Data Availability

The data underlying this article are available upon request to the author.
